# Impact of a 12-week high-intensity interval training program on working memory in young adults with overweight or obesity

**DOI:** 10.3389/fnut.2026.1869229

**Published:** 2026-09-30

**Authors:** Li-Na Zhu, Wei Yuan, Qi-Zheng Gu, Qi-Ling Yang, Chuan-Jie Yi, Zheng Wang

**Affiliations:** College of Physical Education, Yangzhou University, Yangzhou, Jiangsu, China

**Keywords:** high-intensity interval training, maximal oxygen consumption, n-back task, overweight or obesity, working memory, young adults

## Abstract

**Objective:**

To examine the association of a 12-week high-intensity interval training (HIIT) program with working-memory performance in young adults aged 18–25 years with overweight or obesity.

**Methods:**

A total of 120 young adults with overweight or obesity were randomly allocated to an HIIT group (*n* = 48) or a control group (*n* = 72). The HIIT group completed a 12-week structured program, whereas the control group maintained usual daily activities. Working-memory updating was assessed using 1-back and 2-back tasks. Post-test body weight and maximal oxygen consumption (VO_2_max) were analysed using baseline-adjusted analysis of covariance (ANCOVA), whereas body mass index (BMI) was analysed using repeated-measures ANCOVA. Reaction-time outcomes were analysed using a 2 × 2 × 2 mixed repeated-measures ANCOVA, with group as the between-subject factor and time and cognitive load as within-subject factors. Because marked baseline imbalance was observed for accuracy, 1-back and 2-back accuracy were analysed separately using baseline-adjusted post-test ANCOVA. Exploratory partial correlation analyses examined associations between changes in VO_2_max and n-back performance separately within the HIIT and control groups. An additional exploratory linear regression model tested whether the association between VO_2_max improvement and 2-back reaction-time improvement differed between groups.

**Results:**

Baseline-adjusted ANCOVA showed lower post-test body weight in the HIIT group than in the control group [*F*(1,115) = 110.07, *p* < 0.001, η^2^ = 0.256] and higher post-test VO_2_max [*F*(1,115) = 300.86, *p* < 0.001, η^2^ = 0.451]. Repeated-measures ANCOVA showed a significant group × time interaction for BMI [*F*(1,115) = 137.60, *p* < 0.001, η^2^ = 0.545]. For reaction time, the 2 × 2 × 2 mixed repeated-measures ANCOVA showed a significant group × time interaction [*F*(1,115) = 31.95, *p* < 0.001, partial η^2^ = 0.217] and a significant group × cognitive-load interaction [*F*(1,115) = 9.22, *p* = 0.003, partial η^2^ = 0.074], whereas the group × time × cognitive-load interaction was not significant [*F*(1,115) = 2.15, *p* = 0.145, partial η^2^ = 0.018]. After adjustment for baseline accuracy and other measured baseline covariates, no significant post-test group difference was observed in 1-back accuracy [*F*(1,114) = 0.91, *p* = 0.343] or 2-back accuracy [*F*(1,114) = 2.17, *p* = 0.143]. In exploratory analyses, VO_2_max improvement was associated with 2-back reaction-time improvement in the HIIT group (*r* = 0.391, *p* = 0.009) but not in the control group (*r* = −0.101, *p* = 0.411). However, the group × VO_2_max-improvement interaction did not reach conventional statistical significance (β = 388.57, 95% CI: −7.68 to 784.82, *p* = 0.055).

**Conclusion:**

Participation in the 12-week HIIT program was associated with lower adjusted post-test body weight, higher adjusted post-test VO_2_max, a greater reduction in BMI, and more favorable reaction-time performance averaged across n-back loads. No adjusted post-test between-group difference in accuracy was observed. The non-significant group × time × cognitive-load interaction does not support a cognitive-load-specific effect. Although the VO_2_max–2-back reaction-time association was observed in the HIIT group but not in the control group, the non-significant interaction model did not conclusively establish training specificity. Dietary intake, habitual physical activity, sedentary behavior, complete individual-level exercise intensity, and formal assessor blinding were not available; therefore, the observed differences cannot be attributed exclusively to HIIT.

## 1 Introduction

Obesity has become a major public-health concern in the 21st century, with young adults representing a substantially affected population. According to recent estimates, approximately 80.6 million individuals aged 15–24 years were living with obesity worldwide in 2021, and China had the largest population of adults with overweight or obesity, totaling 402 million (1). The Report on Chinese Residents’ Chronic Diseases and Nutrition (2020) indicates that the combined prevalence of overweight and obesity among individuals aged 6–17 years in China has approached 20%. Among adults aged ≥18 years, the prevalence of overweight and obesity has reached 34.3% and 16.4%, respectively (2).

Overweight and obesity have been associated with poorer performance in several executive-function-related domains. Proposed pathways include chronic low-grade inflammation, insulin resistance, vascular alterations, and metabolic disturbances, which may influence brain structure and function (3–10). Executive function is commonly conceptualized as comprising inhibitory control, working-memory updating, and cognitive flexibility (10).

Exercise is a widely used non-pharmacological strategy for improving health outcomes in individuals with overweight or obesity. Evidence suggests that exercise may be associated with favorable cognitive outcomes, potentially through changes in cerebral blood flow, neurotransmitter function, and brain-derived neurotrophic factor (BDNF) (11–14). However, the magnitude and consistency of these associations vary across populations, exercise protocols, and cognitive domains.

Evidence suggests that acute and long-term exercise interventions may be associated with favorable executive-function-related outcomes, although findings vary across studies (15). Exercise modality, duration, intensity, comparator conditions, participant characteristics, and the cognitive domain assessed may contribute to this heterogeneity (16). For example, Amorim et al. reported greater improvement in inhibitory control following 12 weeks of HIIT and self-selected intensity training than following moderate-intensity continuous training (MICT) in adults with overweight or obesity (17). In contrast, evidence for working-memory outcomes is less consistent, and results obtained from one cognitive domain should not be generalized to executive function as a whole.

High-intensity interval training, characterized by alternating short bouts of high-intensity exercise and recovery periods, is time-efficient and may improve cardiorespiratory fitness and adiposity-related outcomes (18, 19). Nevertheless, the cognitive effects of HIIT remain uncertain because previous studies have used heterogeneous samples, intervention protocols, and cognitive tasks (15, 16, 20, 21). Current research has predominantly focused on children and adolescents, whereas studies involving young adults aged 18–25 years with overweight or obesity remain limited and are often characterized by small sample sizes and short intervention durations (22, 23). Young adults differ from children and adolescents in both physiological and cognitive developmental status, limiting the generalizability of existing findings (24, 25).

The present study aimed to examine changes in n-back-indexed working-memory performance following a 12-week HIIT program in young adults with overweight or obesity. We hypothesized that, compared with the control group, the HIIT group would show more favorable changes in reaction time. We also explored changes in accuracy; however, because substantial baseline imbalance was subsequently identified for accuracy, these outcomes were evaluated using baseline-adjusted post-test ANCOVA and interpreted cautiously. In addition, we examined whether changes in VO_2_max were associated with changes in n-back performance.

## 2 Study methods

### 2.1 Study participants

#### 2.1.1 Inclusion criteria

Eligible participants were young adults aged 18–25 years who were currently enrolled as college students and met the criteria for overweight as defined in the Guidelines for Prevention and Control of Overweight and Obesity in Chinese Adults, specifically a body mass index (BMI) of 24.0–31.0 kg/m^2^. Individuals were required to have no history of mental illness, attention deficit hyperactivity disorder, autism spectrum disorder, or other neurodevelopmental disorders, and to demonstrate stable mental status. All participants were required to demonstrate understanding of and compliance with study procedures, provide written informed consent voluntarily, and commit to completing the full intervention and assessment protocol.

#### 2.1.2 Exclusion criteria

Exclusion criteria included comorbid intellectual disability, schizophrenia, other severe psychiatric or neurological disorders, or cognitive impairment. Individuals with contraindications to exercise, including severe cardiopulmonary insufficiency, musculoskeletal disorders, or severe hypertension, were also excluded. Participants were further excluded if they planned to engage in additional exercise programs, weight-loss interventions, or cognitive training during the study period that could influence outcomes. Absence from more than 10% of scheduled exercise sessions, voluntary withdrawal from the study, or loss to follow-up also resulted in exclusion from the final analysis. Regular participation in aerobic exercise, defined as aerobic activity more than once per week or for ≥30 min per session within the preceding 3 months, was not permitted. Exclusion also applied to individuals using weight-loss medications, sedatives, or other psychotropic agents, or those who had previously undergone interventions with potential effects on cognitive function.

No standardized dietary intervention, dietary record, or dietary-intake monitoring was implemented during the 12-week study period. Participants were asked to maintain their usual dietary habits and not to undertake an additional weight-loss program. Accordingly, unmeasured between-group or within-group dietary changes may have contributed to changes in body weight, BMI, VO_2_max, and cognitive performance.

#### 2.1.3 Sample size estimation and grouping

Sample size was calculated using G*Power 3.1 software, with 2-back reaction time designated as the primary outcome. Assuming a significance level (α) of 0.05, statistical power (1 −β) of 0.80, and a moderate effect size (f = 0.25), the minimum required sample size was estimated at 22 participants per group.

A 2:3 HIIT-to-control allocation ratio was used for pragmatic reasons related to instructor capacity, course scheduling, and the number of available participants who could attend the structured intervention. The final sample exceeded the calculated minimum sample size. Baseline characteristics were reported in full, and prespecified baseline covariates were incorporated into the ANCOVA and partial-correlation models.

The study followed CONSORT principles for randomized controlled trials. A total of 175 individuals were assessed for eligibility, 55 of whom did not meet the inclusion criteria. The remaining 120 participants completed baseline assessments. After baseline assessment, an independent third-party researcher who was not involved in recruitment, testing, or intervention delivery generated the allocation sequence using a computer-generated random-number procedure with a prespecified 2:3 HIIT-to-control ratio. Allocation concealment was maintained until completion of baseline assessments using sequentially numbered, opaque, sealed envelopes. No participant was lost to follow-up; thus, 48 HIIT participants (21 males and 27 females) and 72 control participants (39 males and 33 females) were included in the final analysis (Figure 1).

**FIGURE 1:**
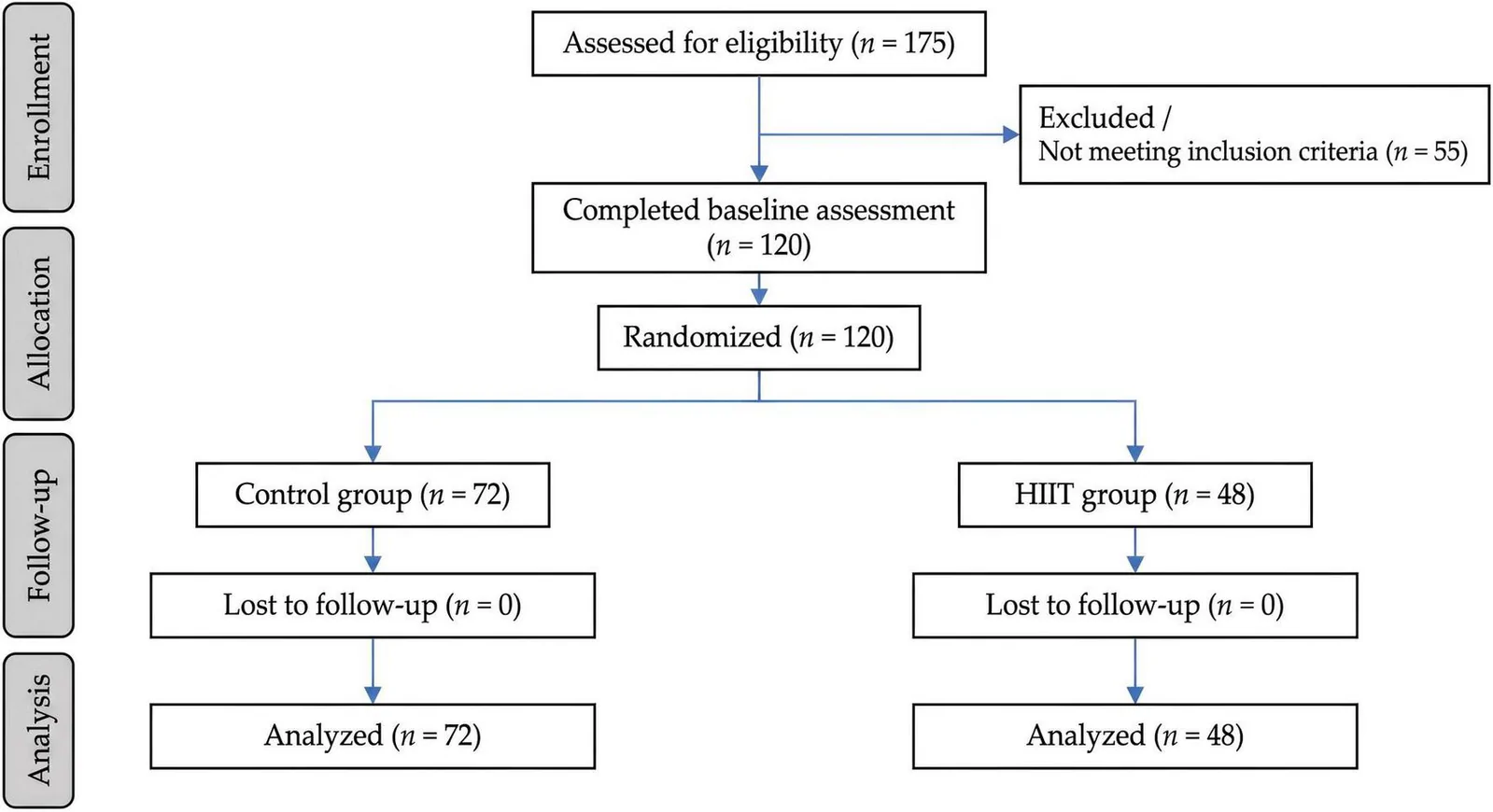
CONSORT flow diagram showing participant screening, random allocation, intervention completion, and final analysis.

Because of the nature of the exercise intervention, participants and intervention instructors were aware of group allocation. Outcome assessors and personnel responsible for data entry were not formally blinded to allocation. To reduce procedural variability, the same standardized assessment protocols, equipment, testing environment, and instructions were used for both groups at baseline and post-test.

### 2.2 Study design

A randomized controlled trial design was employed. The study consisted of three phases: baseline assessment, a 12-week intervention period, and post-intervention assessment. Baseline assessments were conducted within 1 week prior to the intervention and included demographic characteristics, anthropometric measures, cardiorespiratory fitness, and working-memory performance.

Participants in the HIIT group completed a 12-week HIIT program, whereas participants in the control group were instructed to maintain their usual daily activities and received routine follow-up without additional structured exercise. Post-intervention assessments were conducted within 1 week after completion of the intervention using the same outcome measures as at baseline.

### 2.3 Intervention program

#### 2.3.1 Types of exercise

The intervention incorporated a combination of aerobic and anaerobic exercise, calisthenic movement sequences, varied endurance running, and fitness-based games, delivered in an alternating format.

Calisthenic movement sequences consisted of simple, easily acquired combinations of limb movements, in which discrete actions were integrated into structured sequences for repeated practice. These activities required coordinated force production across the upper and lower extremities and the core musculature. Based on the theory of coarticulation, sequence-based movement training may enhance motor coordination (26).

Varied endurance running involved structured route designs to increase engagement, informed by the concept of psychodiversity described by Watkins et al. (27). Dynamic variation in running routes was intended to promote intrinsic motivation while simultaneously imposing additional demands on balance control through frequent directional changes. This approach may enhance neuromuscular coordination and facilitate activation of muscle groups less engaged during linear running (28).

Fitness-based games integrated elements of group collaboration across multiple physical training components. Studies conducted by Gil-Arias et al. and Thi et al. have reported that team-based activities may enhance engagement and physical performance (29, 30).

#### 2.3.2 Exercise intensity

Exercise intensity was prescribed in accordance with HIIT principles, with mean heart rate maintained at ≥85% of maximal heart rate (HRmax) (31). HRmax was estimated using the formula proposed by Tanaka et al. (32):

HR_*max*_ = 208 − (0.7 × age)

During each session, six participants were randomly selected to wear heart-rate monitors (BHT GOFIT) for real-time monitoring. This monitoring strategy was intended to provide session-level verification of exercise intensity rather than complete individual-level heart-rate monitoring for all participants. Exercise intensity was adjusted dynamically based on recorded heart-rate data and ratings of perceived exertion (33). The BHT GOFIT system enabled real-time acquisition of individual heart-rate data for the sampled participants during each session. Complete individual-level heart-rate trajectories across all sessions were not available for every participant.

Rating of perceived exertion (RPE) was also recorded during training using the Borg scale, and participants were encouraged to maintain a perceived exertion level consistent with vigorous-to-high-intensity exercise. Exercise intensity was adjusted when heart-rate or RPE values fell outside the target range.

#### 2.3.3 Exercise frequency and duration

Based on the scheduling characteristics of extracurricular elective courses among college students and the physical tolerance profile of young adults with overweight or obesity, the HIIT intervention was conducted three times per week on non-consecutive days. Each session lasted 60 min, including 40 min of core HIIT training. The intervention period extended over 12 weeks, comprising a total of 36 standardized exercise sessions. This program design was supported by relevant literature (34). The protocol was structured into three phases: warm-up, exercise training, and cool-down.

The 12-week HIIT program was delivered in three progressive 4-week stages. Because participants had overweight or obesity, the program was designed to balance exercise intensity with musculoskeletal safety. During Stages 1 and 2, approximately 60% of the core training content consisted of low-impact movements, including marching, controlled stepping, torso rotation, walking-based recovery, and modified strength-oriented movements. The remaining content included carefully supervised running- and jump-based activities with individualized modification when necessary. During Stage 3, exercise demands were progressively increased through greater movement continuity, running distance, pace, repetition volume, and task complexity while maintaining attention to movement technique and participant tolerance. A detailed standardized weekly intervention schedule, including the content and progression of each exercise component, is provided in Supplementary Table 1.

Warm-up phase (10 min): Low-intensity dynamic activities were used to facilitate physiological preparation, including jogging, joint mobilization, and dynamic stretching. During the initial 3 min, participants jogged at 6–7 km/h, achieving 50%–60% of maximal heart rate (HRmax). This was followed by 4 min of joint mobilization involving the neck, shoulders, elbows, wrists, hips, knees, and ankles (15 repetitions each). The final 3 min consisted of dynamic stretching targeting the lower extremities, core, and upper extremities (2 sets of 10 repetitions).

Exercise training phase (40 min): This phase integrated calisthenic movement sequences, varied endurance running, and fitness-based games in a 15-min + 15-min + 10-min format. Two student assistants supported session implementation, and heart rate was recorded at 5-min intervals to enable timely adjustments in exercise intensity and to ensure both safety and effectiveness.

Calisthenic movement sequences (15 min): Three sets of compound exercises were performed, including burpees, tuck jumps, torso rotations with knee-to-elbow contact, and high knees with under-leg claps, facilitating simultaneous activation of multiple muscle groups through coordinated movement patterns (35). A HIIT protocol consisting of 30 s of high-intensity activity followed by 10 s of rest was implemented (34). Each set lasted 4 min and was followed by a 1-min rest period, for a total of three sets. During Stages 1 and 2, low-impact modified movements accounted for approximately 60% of the calisthenic content; for example, modified burpees without a push-up or jump, step-back movements, controlled squat-based movements, and standing core exercises were used. Tuck jumps and other jump-based actions were introduced or increased only according to participants’ movement technique and tolerance. During Stage 3, repetition volume, movement continuity, and the proportion of standard running- or jump-based actions were progressively increased while maintaining safe landing technique and individualized modification where required.

Varied endurance running (15 min): This component included S-cone slalom running, shuttle running, circular interval running, and group-based chase running. An interval structure of 40 s of fast running followed by 20 s of slow walking was applied, with a 1-min slow-walking recovery period after every five intervals. Evidence indicates that a 1-min recovery interval is favorable for activation of the phosphagen system (36). During Stages 1 and 2, approximately 60% of this component consisted of low-impact locomotor activities, including brisk walking, fast marching, side stepping, controlled directional walking, and slow-walking recovery. The remaining activities included short-distance running tasks at an individually tolerable pace. During Stage 3, running distance, pace, directional-change demands, and route complexity were progressively increased according to the prespecified weekly schedule. Team-based interaction was incorporated throughout this component.

Fitness games (10 min): Group-based activities, including obstacle-course running, plank exercises, and shuttle running, were conducted in accordance with HIIT principles, with three high-intensity segments (18). Based on the HIIT protocol applied in school physical education settings by Domaradzki et al., each segment consisted of 20 s of activity followed by 10 s of rest (37). During Stages 1 and 2, low-impact activities accounted for approximately 60% of game content and included target-reaching, ball-passing while marching or stepping, cone-reaching, plank exercises, and walking-based relay tasks. The remaining activities involved short-distance shuttle running or obstacle-course tasks, with running distance and speed modified according to individual tolerance. During Stage 3, game complexity, movement demands, running distance, and participation intensity were progressively increased according to the prespecified weekly schedule.

Varied endurance running (15 min): This component included S-cone slalom running, shuttle running, circular interval running, and group-based chase running. An interval structure of 40 s of fast running followed by 20 s of slow walking was applied, with a 1-min slow-walking recovery period after every five intervals. Evidence indicates that a 1-min recovery interval is favorable for activation of the phosphagen system (36). Across the intervention stages, locomotor demands were progressively increased through greater running distance, pace, directional-change demands, and/or route complexity according to the prespecified weekly schedule. Team-based interaction was incorporated throughout this component.

Fitness games (10 min): Group-based activities, including obstacle-course running, plank exercises, and shuttle running, were conducted in accordance with HIIT principles, with three high-intensity segments (18). Based on the HIIT protocol applied in school physical education settings by Domaradzki et al., each segment consisted of 20 s of activity followed by 10 s of rest (37). Across the intervention stages, progression was achieved through increasing game complexity, movement demands, and participation intensity according to the prespecified weekly schedule.

Relaxation phase (10 min): Recovery was facilitated primarily through static stretching. During the first 2 min, participants engaged in slow walking to reduce heart rate to <50% HRmax. This was followed by 6 min of static stretching (30 s per movement) combined with controlled abdominal breathing. During the final 2 min, box breathing was performed in accordance with the recovery method described by Kasap et al. (38). A breathing pattern of 4-s inhalation, 2-s breath hold, and 6-s exhalation was applied to promote parasympathetic activation.

#### 2.3.4 Intervention implementation and quality control

The intervention was supervised by physical education instructors with more than 3 years of teaching experience and supported by two student assistants. Attendance and exercise duration were systematically recorded throughout the intervention period. Heart rate was monitored in a randomly selected subset of six HIIT participants during each session to provide session-level verification of exercise intensity; complete individual-level heart-rate data were not available for all participants across the intervention period. Participant feedback was collected, and exercise intensity was adjusted based on RPE (39). Participants in the control group were followed up at 4-week intervals and maintained their usual daily activities without additional structured intervention.

### 2.4 Measurement indicators and testing methods

#### 2.4.1 Working memory assessment

The n-back paradigm was used to assess working memory updating, a core component of executive function (40). Inhibitory control and cognitive flexibility were not assessed in the present study. The n-back paradigm requires participants to compare the current stimulus with the n^th^ previous stimulus. The working memory load is manipulated by controlling the number of stimuli between the current stimulus and the target stimulus. The experimental task was programmed using the PsychoPy software. Uppercase letters were presented in a pseudorandomized order across trials (41). Each stimulus was displayed for 1,000 ms, with a stimulus-onset asynchrony of 3,000 ms. In the 1-back condition, participants judged whether the current letter matched the immediately preceding letter; in the 2-back condition, they judged whether the current letter matched the letter presented two trials earlier. For both conditions, participants pressed the “F” key for a match and the “J” key for a non-match.

The formal assessment consisted of two blocks of 25 trials for each condition (50 trials per condition). Accuracy was calculated as the proportion of correct responses among all scored trials. Before the formal test, participants completed a 12-trial practice session. The experimenter confirmed that each participant could recognize the letter stimuli, understood the response rules, and was familiar with the key-press procedure before the formal task began. No formal practice-accuracy threshold was prespecified.

All assessments were performed in a quiet and well-lit computer laboratory. Participants were instructed to maintain a regular routine and to avoid consumption of stimulant beverages prior to testing.

#### 2.4.2 Aerobic exercise capacity test

Maximal oxygen consumption (VO_2_max) was assessed using an incremental exercise protocol as an indicator of aerobic capacity (42). Participants performed graded exercise on an Elmmed EGT1000 cycle ergometer, while respiratory gas exchange was measured using a Cortex Metalyzer II desktop metabolic system (Germany).

The protocol commenced with an initial workload of 50 W, with cadence maintained at 55–60 revolutions per minute (rpm). Workload was increased by 50 W every 3 min until volitional exhaustion was reached. Prior to testing, participants completed the Physical Activity Readiness Questionnaire (PAR-Q) to ensure the absence of recent high-intensity exercise and the non-use of medications or substances that could affect neural activity (43).

A 3 to 5 min warm-up was performed, and baseline heart rate (BHR) was recorded. Exercise testing commenced after heart rate had returned to resting levels. During the test, heart rate was continuously monitored using a Polar heart rate monitor. VO_2_max attainment was determined according to established criteria, with achievement confirmed when at least two to three of the following conditions were met (44): (1) oxygen uptake plateaued or decreased despite increasing workload (1500 ml/min); (2) respiratory exchange ratio exceeded 1.1; (3) heart rate exceeded 90% of age-predicted maximum heart rate; and (4) inability to maintain the required cadence of 55–60 rpm despite verbal encouragement. Upon completion of the test, VO_2_max values were recorded. Participants then rested for approximately 5 min and were discharged only after confirmation that no adverse reactions had occurred.

#### 2.4.3 Demographic characteristics and basic physical indicators

Demographic data, including sex and age, as well as anthropometric measures, including height and body weight, were collected. Height was measured using a stadiometer (accuracy: 0.01 cm), and body weight was measured using an electronic scale (accuracy: 0.01 kg). BMI was calculated as body weight (kg) divided by height squared (m^2^).

### 2.5 Statistical analysis

Statistical analyses were conducted using SPSS version 20.0. A total of 120 young adults with overweight or obesity who completed the full intervention and assessment protocol were included in the final analysis. The control group consisted of 72 participants (39 males and 33 females), and the HIIT group consisted of 48 participants (21 males and 27 females). No attrition occurred in either group.

Independent-samples *t*-tests were used to compare baseline characteristics between groups. For post-test body weight, baseline-adjusted ANCOVA was conducted with post-test body weight as the dependent variable, group as the fixed factor, and baseline body weight, baseline height, and baseline VO_2_max as covariates. For post-test VO_2_max, baseline-adjusted ANCOVA was conducted with post-test VO_2_max as the dependent variable, group as the fixed factor, and baseline VO_2_max, baseline height, and baseline body weight as covariates.

Body mass index was analysed using repeated-measures ANCOVA, with group (HIIT vs. control) as the between-subject factor and time (pre-test vs. post-test) as the within-subject factor. Baseline height, body weight, and VO_2_max were included as covariates.

Reaction-time data were analysed using a 2 × 2 × 2 mixed repeated-measures ANCOVA, with group (HIIT vs. control) as the between-subject factor and time (pre-test vs. post-test) and cognitive load (1-back vs. 2-back) as within-subject factors. Baseline height, body weight, and VO_2_max were included as covariates. Significant interaction effects were followed by Bonferroni-adjusted pairwise comparisons.

Because marked baseline differences were observed in 1-back and 2-back accuracy, these outcomes were analysed separately using baseline-adjusted ANCOVA. In each model, post-test accuracy was entered as the dependent variable, group was entered as the fixed factor, and the corresponding baseline accuracy score, baseline height, baseline body weight, and baseline VO_2_max were included as covariates.

Model assumptions were assessed using residual normality and homogeneity-of-variance tests. Where assumptions were not fully met, non-parametric tests were conducted as sensitivity analyses for between-group comparisons. Effect sizes are reported as eta squared (η^2^) or partial eta squared (partial η^2^), as appropriate for the model output, and Cohen’s d for baseline between-group comparisons. A Bonferroni-adjusted threshold of *p* < 0.0125 was applied across the four cognitive outcomes.

Exploratory partial correlation analyses were conducted separately in the HIIT group (*n* = 48) and the control group (*n* = 72) to examine associations between changes in VO_2_max and changes in each n-back outcome. Each model was adjusted for baseline height, baseline body weight, baseline VO_2_max, and the corresponding baseline cognitive outcome. Two-sided 95% confidence intervals were estimated using 1,000 bootstrap replicates. A Bonferroni-adjusted threshold of *p* < 0.0125 was applied across the four correlations within each group.

To examine whether the association between VO_2_max improvement and 2-back reaction-time improvement differed between groups, an additional exploratory linear regression model was fitted with 2-back reaction-time improvement as the dependent variable. Predictors included VO_2_max improvement, group, the VO_2_max improvement × group interaction, baseline height, baseline body weight, baseline VO_2_max, and baseline 2-back reaction time. The interaction term was used to test whether the association differed between groups. All correlation and interaction analyses were exploratory and were not designed to establish mediation or causality.

## 3 Results

### 3.1 Baseline characteristics of study participants

Baseline characteristics are presented in Table 1. No statistically significant between-group differences were observed in age [*t*(118) = −1.45, *p* = 0.149, Cohen’s *d* = −0.26], BMI [*t*(118) = −0.271, *p* = 0.787, Cohen’s *d* = −0.050], 1-back reaction time [*t*(118) = −1.285, *p* = 0.201, Cohen’s *d* = −0.240], or 2-back reaction time [*t*(118) = 0.017, *p* = 0.986, Cohen’s *d* = 0.003].

**TABLE 1:** Baseline characteristics of study participants at pre-intervention (mean ± standard deviation).

Characteristics	Control group (*n* = 72)	HIIT Intervention group (*n* = 48)	*t*-value	*P*-value	Cohen’s d
Sex (male/female, *n*)	39/33	21/27	–	–	–
Age (years)	18.500 ± 0.690	18.670 ± 0.560	−1.450	0.149	- 0.260
Height (m)	1.698 ± 0.084	1.656 ± 0.082	2.735	0.007	0.510
Weight (kg)	86.569 ± 9.205	82.670 ± 10.970	2.104	0.037	0.392
BMI (kg/m^2^)	29.964 ± 1.691	30.060 ± 1.970	−0.271	0.787	−0.050
VO_2_max (L/min)	1.261 ± 0.369	1.080 ± 0.360	2.587	0.011	0.482
Reaction time under the 1-back condition (ms)	810.645 ± 281.328	877.810 ± 279.052	−1.285	0.201	−0.240
Reaction time under the 2-back condition (ms)	1189.855 ± 170.656	1189.257 ± 208.119	0.017	0.986	0.003
Accuracy under the 1-back condition	0.939 ± 0.056	0.810 ± 0.170	5.998	<0.001	1.118
Accuracy under the 2-back condition	0.836 ± 0.081	0.765 ± 0.053	5.386	<0.001	1.004

However, the control group had greater baseline height [*t*(118) = 2.735, *p* = 0.007, Cohen’s *d* = 0.510], body weight [*t*(118) = 2.104, *p* = 0.037, Cohen’s *d* = 0.392], and VO_2_max [*t*(118) = 2.587, *p* = 0.011, Cohen’s *d* = 0.482] than the HIIT group. The control group also had higher baseline 1-back accuracy [*t*(118) = 5.998, *p* < 0.001, Cohen’s *d* = 1.118] and 2-back accuracy [*t*(118) = 5.386, *p* < 0.001, Cohen’s *d* = 1.004]. These baseline differences were considered in the adjusted analyses.

### 3.2 Exercise intensity manipulation test

During the intervention period, the mean heart rate recorded during high-intensity intervals was 176.641 ± 7.773 beats per minute, corresponding to approximately 87% of HRmax. In comparison, mean heart rates during the warm-up phase (82.856 ± 10.74 bpm) and cool-down phase (111.067 ± 10.137 bpm) were substantially lower.

Repeated-measures ANOVA indicated a statistically significant difference across phases [F (2, 105) = 280.592, *p* < 0.001, partial *η^2^* = 0.842]. Among the randomly monitored participants, the mean heart rate during the high-intensity intervals corresponded to approximately 87% of HRmax, which was consistent with the planned HIIT intensity criterion. However, because heart rate was monitored only in a randomly selected subset of six participants during each session, these data provide session-level intensity verification and cannot confirm that every participant achieved the target intensity in every session.

### 3.3 Physiological changes and effects of the physical fitness intervention

Because baseline differences were observed in body weight and VO_2_max, these outcomes were analysed using separate baseline-adjusted ANCOVA models, with the post-test value as the dependent variable and group as the fixed factor. For body weight, baseline body weight, height, and baseline VO_2_max were included as covariates. For VO_2_max, baseline VO_2_max, height, and baseline body weight were included as covariates. BMI was analysed separately using repeated-measures ANCOVA, with group as the between-subject factor and time as the within-subject factor.

For body weight, the adjusted group effect at post-test was statistically significant, *F*(1,115) = 110.073, *p* < 0.001, η^2^ = 0.256 (95% CI: 0.130–0.380). After adjustment for baseline body weight, height, and baseline VO_2_max, the HIIT group had lower post-test body weight than the control group. The adjusted mean difference (HIIT minus control) was −8.305 kg (95% CI: −9.873 to −6.737; SE = 0.792; *t*(115) = −10.492; *p* < 0.001; Cohen’s *d* = −2.034). Baseline body weight was significantly associated with post-test body weight [*F*(1,115) = 204.738, *p* < 0.001, η^2^ = 0.475], whereas height [*F*(1,115) = 0.008, *p* = 0.931] and baseline VO_2_max [*F*(1,115) = 0.875, *p* = 0.351] were not significant covariates. Descriptively, body weight remained stable in the control group (86.57 ± 9.21 kg at pre-test and 86.49 ± 9.04 kg at post-test), whereas it decreased in the HIIT group (82.67 ± 10.97 kg at pre-test and 74.15 ± 12.93 kg at post-test). Non-parametric sensitivity analysis also supported a between-group difference at post-test (Kruskal–Wallis statistic = 37.596, *p* < 0.001).

For BMI, repeated-measures ANCOVA showed no significant main effect of time, *F*(1,115) = 1.829, *p* = 0.179, η^2^ = 0.016. However, the group × time interaction was statistically significant, *F*(1,115) = 137.604, *p* < 0.001, η^2^ = 0.545. Bonferroni-adjusted comparisons showed no between-group difference at pre-test (mean difference = 0.044 kg/m^2^, 95% CI: −0.372 to 0.461, *p*_*Bonf*_ = 0.834), whereas BMI was lower in the HIIT group than in the control group at post-test (mean difference = 3.418 kg/m^2^, 95% CI: 3.001–3.834, *p_*Bonf*_* < 0.001). Descriptively, BMI changed minimally in the control group (29.96 ± 1.69 to 29.80 ± 1.66 kg/m^2^) but decreased in the HIIT group (30.06 ± 1.98 to 26.39 ± 2.99 kg/m^2^).

For VO_2_max, the baseline-adjusted ANCOVA showed a statistically significant group effect at post-test, *F*(1,115) = 300.860, *p* < 0.001, η^2^ = 0.451 (95% CI: 0.321–0.557). After adjustment for baseline VO_2_max, height, and baseline body weight, post-test VO_2_max was higher in the HIIT group than in the control group. The adjusted mean difference (HIIT minus control) was 0.557 L/min (SE = 0.032; *t*(115) = 17.345; *p* < 0.001; Cohen’s *d* = 3.362). Baseline VO_2_max was significantly associated with post-test VO_2_max [*F*(1,115) = 245.704, *p* < 0.001, η^2^ = 0.368]. Baseline body weight showed a small association with post-test VO_2_max [*F*(1,115) = 5.961, *p* = 0.016, η^2^ = 0.009], whereas height was not significantly associated with post-test VO_2_max [*F*(1,115) = 0.031, *p* = 0.860]. Descriptively, VO_2_max changed minimally in the control group (1.261 ± 0.369 to 1.290 ± 0.350 L/min) but increased in the HIIT group (1.084 ± 0.361 to 1.687 ± 0.343 L/min).

Overall, baseline-adjusted ANCOVA showed lower post-test body weight and higher post-test VO_2_max in the HIIT group than in the control group, whereas repeated-measures ANCOVA demonstrated a significant group × time interaction for BMI (see Figure 2). Although these findings are consistent with favorable physiological changes in the HIIT group, dietary intake, habitual physical activity, sedentary behavior, and complete individual-level training intensity were not quantitatively monitored. Therefore, the observed differences cannot be attributed exclusively to HIIT.

**FIGURE 2:**
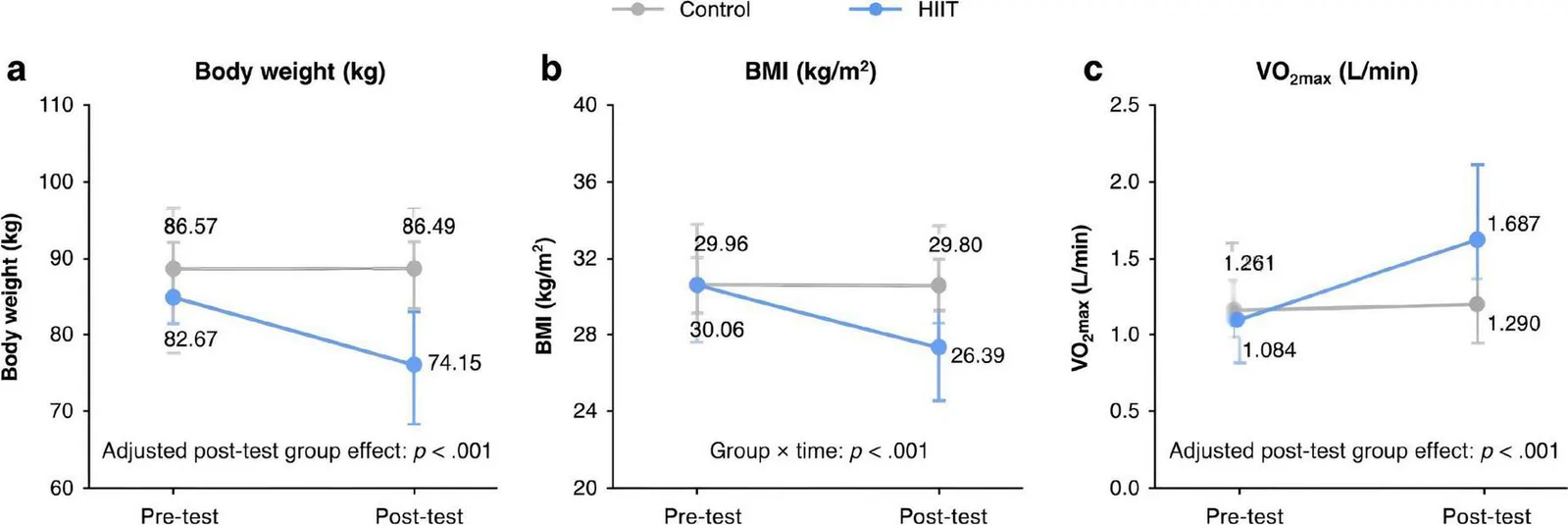
Physiological outcomes before and after the 12-week intervention. **(a)** Body weight; **(b)** BMI; **(c)** VO_2max_. Points represent unadjusted group means, and error bars represent 95% confidence intervals. Post-test body weight and VO_2max_ were analysed using baseline-adjusted ANCOVA, with relevant baseline covariates included in each model. BMI was analysed using repeated-measures ANCOVA with group and time as factors. The HIIT group had lower adjusted post-test body weight and higher adjusted post-test VO_2max_ than the control group (both *p* < 0.001). BMI showed a significant group × time interaction (*p* < 0.001).

### 3.4 Working-memory performance on the n-back task

At baseline, reaction time did not differ significantly between groups in either the 1-back or 2-back condition. In contrast, the control group had substantially higher baseline accuracy than the HIIT group in both conditions. Therefore, accuracy outcomes were analysed separately using baseline-adjusted ANCOVA (see Figure 3).

**FIGURE 3:**
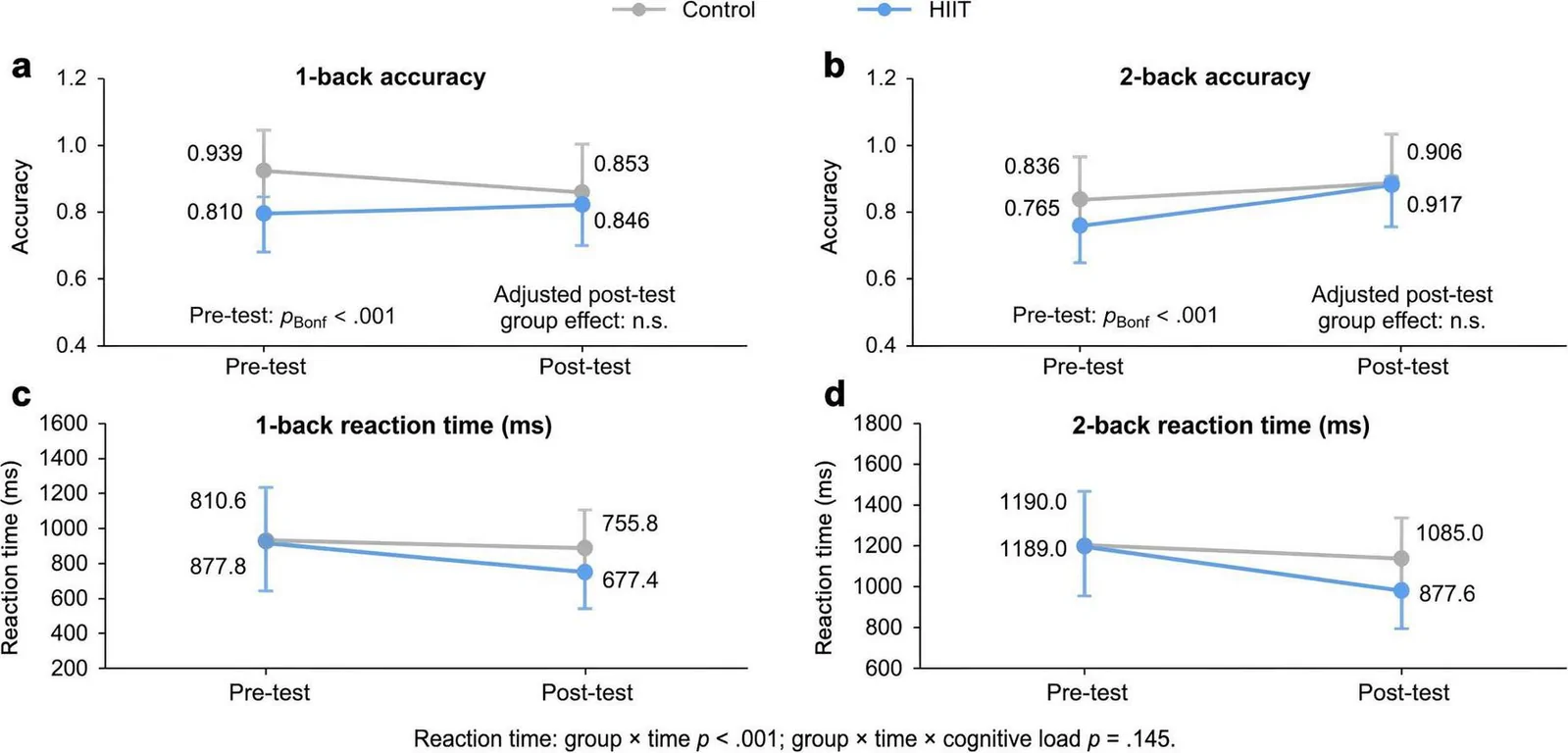
N-back task performance before and after the 12-week intervention in the control and HIIT groups. Panels show **(a)** 1-back accuracy, **(b)** 2-back accuracy, **(c)** 1-back reaction time, and **(d)** 2-back reaction time. Points represent unadjusted group means, error bars represent 95% confidence intervals, and connecting lines depict descriptive pre-to-post trajectories. Accuracy was analysed separately for each load condition using baseline-adjusted post-test ANCOVA, with the corresponding baseline accuracy score, height, body weight, and baseline VO_2_max as covariates. Adjusted analyses showed no significant post-test between-group difference in 1-back or 2-back accuracy. Reaction-time data were analysed using a 2 × 2 × 2 mixed repeated-measures ANCOVA with group as the between-subject factor and time and cognitive load as within-subject factors, adjusted for baseline height, body weight, and baseline VO_2_max. The group × time interaction was significant, whereas the group × time × cognitive-load interaction was not significant; therefore, the figure does not demonstrate a cognitive-load-specific effect.

For 1-back accuracy, the adjusted post-test difference between groups was not statistically significant [*F*(1,114) = 0.906, *p* = 0.343, η^2^ = 0.008]. The adjusted contrast for HIIT relative to control was 0.023 (95% CI: −0.025 to 0.070). For 2-back accuracy, the adjusted post-test difference was also not statistically significant [*F*(1,114) = 2.174, *p* = 0.143, η^2^ = 0.017]. The adjusted contrast for HIIT relative to control was 0.029 (95% CI: −0.010 to 0.067). Non-parametric sensitivity analyses were consistent with these results for 1-back accuracy (Kruskal–Wallis statistic = 0.575, *p* = 0.448) and 2-back accuracy (Kruskal–Wallis statistic = 0.062, *p* = 0.803).

Accordingly, after adjustment for the corresponding baseline accuracy score, height, body weight, and baseline VO_2_max, there was no evidence of a post-test between-group difference in 1-back or 2-back accuracy. The convergence of unadjusted group means should be interpreted descriptively and does not demonstrate an intervention-related improvement in accuracy.

Reaction-time data were analysed using a 2 × 2 × 2 mixed repeated-measures ANCOVA, with group as the between-subject factor and time and cognitive load as within-subject factors; baseline height, body weight, and VO_2_max were included as covariates. There was no significant main effect of time [*F*(1,115) = 0.170, *p* = 0.681, partial η^2^ = 0.001] or cognitive load [*F*(1,115) = 0.661, *p* = 0.418, partial η^2^ = 0.006]. A significant group × time interaction was observed [*F*(1,115) = 31.950, *p* < 0.001, partial η^2^ = 0.217], indicating that reaction-time changes differed between groups. A significant group × cognitive-load interaction was also observed [*F*(1,115) = 9.218, *p* = 0.003, partial η^2^ = 0.074]. The time × cognitive-load interaction was not significant [*F*(1,115) = 2.673, *p* = 0.105, partial η^2^ = 0.023], and the group × time × cognitive-load interaction was not significant [*F*(1,115) = 2.153, *p* = 0.145, partial η^2^ = 0.018].

Bonferroni-adjusted comparisons averaged across cognitive-load conditions showed no between-group difference at pre-test (mean difference = −18.350 ms, SE = 36.112, *p*_*Bonf*_ = 1.000), whereas the HIIT group had faster reaction time than the control group at post-test (mean difference = 159.446 ms, SE = 36.112, *p*_*Bonf*_ < 0.001). Within-group comparisons showed a reduction in reaction time from pre-test to post-test in the HIIT group (mean difference = 256.877 ms, SE = 23.992, *p*_*Bonf*_ < 0.001) and a smaller reduction in the control group (mean difference = 79.081 ms, SE = 19.435, *p*_*Bonf*_ < 0.001).

For descriptive purposes, 1-back reaction time changed from 810.65 ± 281.33 ms to 755.78 ± 250.38 ms in the control group and from 877.81 ± 279.05 ms to 677.41 ± 203.30 ms in the HIIT group. Two-back reaction time changed from 1189.86 ± 170.66 ms to 1085.43 ± 203.95 ms in the control group and from 1189.26 ± 208.12 ms to 877.59 ± 227.75 ms in the HIIT group. A load-specific post-test comparison showed faster 2-back reaction time in the HIIT group than in the control group (mean difference = 382.29 ms, 95% CI: 320.36–444.22, *p* < 0.001). However, because the group × time × cognitive-load interaction was not significant, this follow-up comparison should not be interpreted as evidence of a cognitive-load-specific or 2-back-specific intervention effect.

### 3.5 Exploratory associations between VO_2_max improvement and changes in working-memory performance

Exploratory partial correlation analyses were conducted separately in the HIIT group (*n* = 48) and the control group (*n* = 72). The analyses adjusted for baseline height, baseline body weight, baseline VO_2_max, and the corresponding baseline cognitive outcome. Two-sided 95% confidence intervals were estimated using 1,000 bootstrap replicates. A Bonferroni-adjusted threshold of *p* < 0.0125 was applied across the four correlations within each group.

In the HIIT group, VO_2_max improvement was not significantly associated with changes in 1-back accuracy after Bonferroni correction (*r* = 0.298, unadjusted *p* = 0.049, 95% CI: 0.029–0.518). No significant associations were observed for changes in 2-back accuracy (*r* = 0.166, *p* = 0.282, 95% CI: −0.144 to 0.397) or 1-back reaction time (*r* = −0.060, *p* = 0.698, 95% CI: −0.368 to 0.270). VO_2_max improvement was positively associated with 2-back reaction-time improvement (*r* = 0.391, *p* = 0.009, 95% CI: 0.098–0.613), and this association remained statistically significant after Bonferroni correction.

In the control group, VO_2_max improvement was not significantly associated with changes in 1-back accuracy (*r* = −0.071, *p* = 0.567, 95% CI: −0.308 to 0.109), 2-back accuracy (*r* = 0.028, *p* = 0.822, 95% CI: −0.225 to 0.275), 1-back reaction time (*r* = 0.074, *p* = 0.548, 95% CI: −0.178 to 0.303), or 2-back reaction time (*r* = −0.101, *p* = 0.411, 95% CI: −0.321 to 0.141). None of the control-group associations met the Bonferroni-adjusted threshold. Control-group scatterplots are presented in Supplementary Figure 1.

To further examine whether the association between VO_2_max improvement and 2-back reaction-time improvement differed by group, an exploratory linear regression model was fitted with 2-back reaction-time improvement as the dependent variable. The model included VO_2_max improvement, group, their interaction term, baseline height, baseline body weight, baseline VO_2_max, and baseline 2-back reaction time. The group × VO_2_max-improvement interaction did not reach conventional statistical significance (β = 388.57, 95% CI: −7.68 to 784.82, *p* = 0.055), although its direction suggested a stronger positive association in the HIIT group than in the control group (see Figure 4). Therefore, the present data do not conclusively establish that the observed HIIT-group association was training-specific. All correlation and interaction analyses were exploratory and do not establish mediation or causality.

**FIGURE 4:**
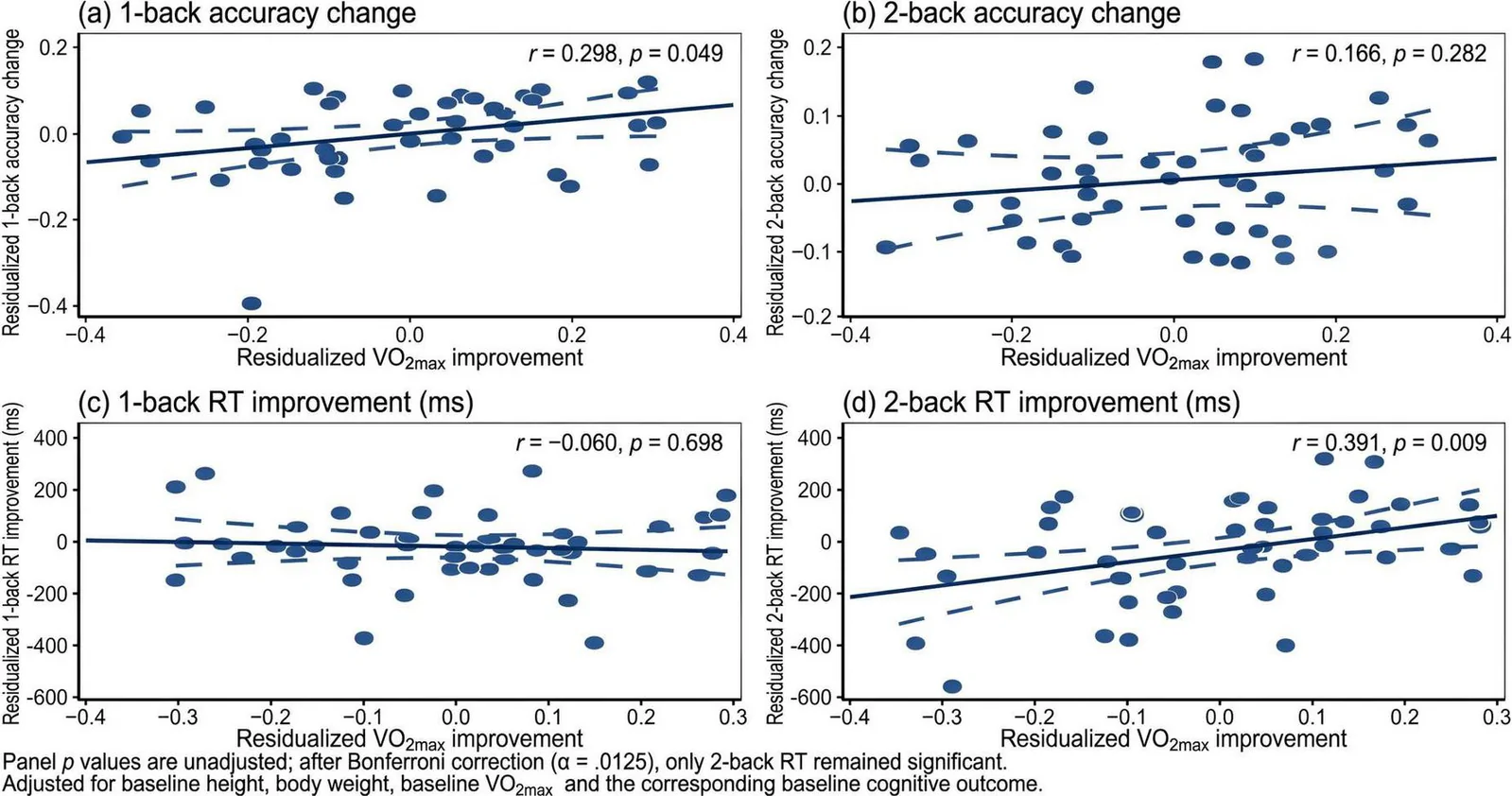
Exploratory partial correlations between VO_2_max improvement and changes in n-back task performance in the HIIT group (n = 48). Analyses were adjusted for baseline height, body weight, baseline VO_2_max, and the corresponding baseline cognitive outcome. Panels show associations between VO_2_max improvement and changes in panel **(a)** 1-back accuracy, **(b)** 2-back accuracy, **(c)** 1-back reaction time, and **(d)** 2-back reaction time. Reaction-time improvement was calculated as pre-test minus post-test reaction time; thus, positive values indicate greater reductions in reaction time. Solid lines indicate fitted regression lines, and dashed lines indicate 95% confidence bands. The reported *p*-values are unadjusted; after Bonferroni correction for four correlations within the HIIT group (α = 0.0125), only the association with 2-back reaction-time improvement remained statistically significant. Parallel control-group analyses are shown in Supplementary Figure 1. An additional exploratory regression interaction analysis did not demonstrate a statistically significant difference between groups in the VO_2_max-2-back reaction-time association (*p* = 0.055). These analyses do not establish training specificity or causality.

## 4 Discussion

Current exercise-based interventions for individuals with overweight or obesity are generally categorized into aerobic training, resistance training, and combined training integrating both approaches (45–47). Moderate-intensity continuous training (MICT) is widely used but may be relatively time-intensive, whereas resistance training is important for preserving lean mass during weight management (48). Combined exercise approaches have therefore been recommended in guidelines for comprehensive obesity management (45). Within this context, high-intensity interval training (HIIT) has attracted increasing interest because it can be delivered in a time-efficient format and may improve cardiorespiratory fitness and adiposity-related outcomes (49, 50).

Previous evidence suggests that HIIT can produce cardiorespiratory-fitness and body-composition outcomes comparable to, or in some settings greater than, those of traditional aerobic training (51, 52). However, the cognitive literature remains heterogeneous because studies differ in participant characteristics, intervention protocols, comparator conditions, and the cognitive domains assessed. In particular, evidence from one task or one executive-function-related domain should not be generalized to executive function as a whole. The present study therefore focused specifically on working-memory updating measured using 1-back and 2-back tasks in young adults with overweight or obesity.

After adjustment for baseline covariates, the HIIT group had lower post-test body weight and higher post-test VO_2_max than the control group. BMI also showed a significant group × time interaction, with a greater reduction in the HIIT group. These findings are consistent with previous reports that HIIT may be associated with favorable changes in body mass, adiposity-related outcomes, and cardiorespiratory fitness (50–52). Nevertheless, body weight and VO_2_max were evaluated using baseline-adjusted post-test ANCOVA rather than a group × time model. Accordingly, these findings should be described as adjusted post-test group differences rather than definitive intervention-related change effects. In addition, dietary intake, habitual physical activity, sedentary behavior, and complete individual-level exercise intensity were not quantitatively monitored; therefore, the observed physiological differences cannot be attributed exclusively to HIIT.

The reaction-time findings indicated a significant group × time interaction in the mixed ANCOVA model, suggesting that changes in reaction time differed between the HIIT and control groups across the intervention period. Averaged across the two n-back load conditions, the HIIT group showed faster post-test reaction time than the control group and a larger pre-to-post reduction in reaction time. Although a load-specific follow-up comparison showed faster 2-back reaction time in the HIIT group at post-test, the group × time × cognitive-load interaction was not significant. Therefore, the available data do not support a cognitive-load-specific or 2-back-specific intervention effect, and the load-specific comparison should be interpreted only as a follow-up finding.

In contrast, no adjusted post-test between-group difference was observed for either 1-back or 2-back accuracy. The control group had substantially higher accuracy at baseline in both conditions, with large standardized between-group differences. Although baseline-adjusted ANCOVA was used, the convergence of the unadjusted group means at post-test does not demonstrate that HIIT improved accuracy. Regression to the mean and residual baseline confounding remain plausible explanations for part of the observed descriptive pattern. Therefore, the present findings do not support a conclusion that HIIT improved working-memory accuracy. This distinction is important because the n-back task reflects working-memory updating only and does not provide a comprehensive assessment of executive function.

The present study used a 2:3 HIIT-to-control allocation ratio because of practical constraints related to instructor capacity, elective-course scheduling, and the number of participants able to attend the structured intervention. Although the final sample exceeded the *a priori* minimum sample-size requirement, unequal allocation is statistically less efficient than 1:1 allocation for a fixed total sample size and may increase the probability of chance baseline imbalance. In the present study, baseline differences were observed for height, body weight, VO_2_max, and n-back accuracy. These differences may reflect chance imbalance under the unequal allocation design and individual heterogeneity in this university population; however, they also increase the risk that observed between-group differences are affected by residual confounding. Baseline covariates were included in the relevant ANCOVA and partial-correlation models, but statistical adjustment cannot fully eliminate bias arising from substantial baseline imbalance. Future trials should use 1:1 allocation where feasible and should consider stratified or minimization randomization for important baseline variables.

The intervention was implemented through calisthenic movement sequences, varied endurance running, and fitness-based games. This diversified format may be practically feasible within extracurricular university exercise settings and may be more engaging than repetitive exercise routines. No participant was lost to follow-up; however, the absence of attrition should not be interpreted as direct evidence of high adherence, acceptability, or uniform intervention exposure. In the randomly monitored subset, mean heart rate during high-intensity intervals corresponded to approximately 87% of predicted HRmax, which was consistent with the planned intensity criterion. However, heart-rate monitoring was conducted in only six randomly selected HIIT participants during each session, and complete individual-level heart-rate trajectories were unavailable. Thus, these data provide session-level intensity verification but cannot establish that every participant achieved the intended intensity in every session. The findings of Amorim et al. (17) similarly indicate that exercise interventions may be associated with cognitive outcomes in adults with overweight or obesity, but differences in cognitive tasks, exercise protocols, participant characteristics, and baseline balance limit direct comparison.

The exploratory partial-correlation analyses showed that greater improvement in VO_2_max was associated with greater improvement in 2-back reaction time within the HIIT group after adjustment for measured baseline covariates. This association remained statistically significant after Bonferroni correction. No corresponding significant association was observed in the control group. However, a significant association in one group and a non-significant association in another group do not by themselves demonstrate a statistically significant difference between groups. In the additional interaction model, the group × VO_2_max-improvement term did not reach conventional statistical significance (*p* = 0.055). Therefore, although the overall pattern was consistent with a stronger association in the HIIT group, the present data do not conclusively establish training specificity. The analyses were exploratory and did not include a mediation model; consequently, they cannot establish that improved VO_2_max mediates, explains, or causes the reaction-time findings.

The present study did not measure inflammatory, metabolic, neurotrophic, cerebrovascular, neurophysiological, or neuroimaging markers. Accordingly, the observed exploratory association between VO_2_max improvement and 2-back reaction-time improvement cannot be attributed to a specific biological mechanism. Previous literature linking obesity-related metabolic dysregulation with brain structure, perfusion, insulin action, and cognition (6, 7, 53–55) provides background context only and should not be interpreted as evidence for mechanisms operating in the present sample. Future studies should prospectively measure relevant biological markers and use adequately powered longitudinal designs to evaluate potential pathways.

Several limitations should be acknowledged. First, the pragmatic 2:3 allocation was less statistically efficient than 1:1 allocation and may have increased the likelihood of chance baseline imbalance. This concern is particularly important for accuracy outcomes because the control group had substantially higher baseline 1-back and 2-back accuracy, with large standardized differences. Although baseline-adjusted analyses were conducted, regression to the mean and residual confounding cannot be excluded. Future trials should use balanced allocation where feasible and consider stratified or minimization randomization based on key prognostic variables.

Second, the usual-activity control group did not control for attention, social interaction, participant expectations, or the non-specific effects of structured exercise. Accordingly, the present study was not designed to determine whether HIIT produces effects distinct from those of moderate-intensity continuous training (MICT) or another active exercise comparator. Participants and intervention instructors could not be blinded because of the nature of the intervention, and outcome assessors and data-entry personnel were not formally blinded; therefore, performance and detection bias cannot be excluded. Future trials should include an intensity- and contact-matched MICT or other active exercise comparator, use blinded outcome assessors and data coding, and, where feasible, conduct blinded statistical analyses.

Third, dietary intake, habitual physical activity, sedentary behavior, non-study exercise, caffeine intake, meal timing, and sleep-related behaviors were not continuously or quantitatively monitored. These factors may have influenced anthropometric, cardiorespiratory, and cognitive outcomes. Future studies should use dietary records or standardized dietary guidance, wearable devices to quantify activity and sedentary time, and standardized pre-assessment restrictions.

Fourth, heart rate was monitored only in a randomly selected subset of six HIIT participants per session. Therefore, the data provide session-level verification but cannot confirm individual exercise dose or target-intensity attainment across all sessions. Although the intervention used a prespecified three-stage progression, future studies should collect continuous individual-level heart-rate data, attendance records, session-RPE data, and detailed progression metrics for all participants.

Fifth, the n-back task assessed working-memory updating only and did not assess inhibitory control, cognitive flexibility, or other cognitive domains. The findings should therefore not be generalized to executive function as a whole. Finally, no peripheral or central biological markers were collected. The observed VO_2_max–reaction-time association should not be interpreted as mechanistic, training-specific, or causal. Future studies should include a broader cognitive battery, relevant biomarkers, and appropriately powered longitudinal analyses.

In summary, participation in the 12-week HIIT program was associated with lower adjusted post-test body weight, higher adjusted post-test VO_2_max, a greater reduction in BMI, and more favorable reaction-time performance averaged across n-back loads. No adjusted post-test between-group difference in n-back accuracy was observed. The exploratory association between VO_2_max improvement and 2-back reaction-time improvement was observed in the HIIT group but not in the control group. However, the group × VO_2_max-improvement interaction did not reach conventional statistical significance; therefore, the findings do not conclusively establish training specificity or a causal pathway. Interpretation should remain cautious because of unequal allocation, baseline imbalance, lack of an active exercise comparator, and formal assessor blinding, and unmeasured behavioral and intervention-dose-related factors.

## 5 Conclusion

In young adults with overweight or obesity, participation in the 12-week HIIT program was associated with lower adjusted post-test body weight, higher adjusted post-test VO_2_max, and a greater reduction in BMI. Reaction-time changes differed between groups when averaged across the two n-back load conditions, whereas no adjusted post-test between-group difference was observed for accuracy. Because the group × time × cognitive-load interaction was not significant, the findings do not support a cognitive-load-specific or 2-back-specific effect. The exploratory association between VO_2_max improvement and 2-back reaction-time improvement was statistically significant in the HIIT group but not in the control group; however, the group × VO_2_max-improvement interaction did not reach conventional statistical significance. Therefore, the present data do not conclusively establish training specificity, mediation, or causality. Interpretation is limited by unequal allocation, baseline imbalance, the usual-activity control without an active exercise comparator, absence of formal assessor blinding, unmeasured diet and habitual activity, and incomplete individual-level heart-rate monitoring.

## Data Availability

The original contributions presented in this study are included in this article/Supplementary material, further inquiries can be directed to the corresponding author.

## Supplementary material

Supplementary Figure 1 Exploratory partial correlations between VO_2_max improvement and changes in n-back task performance in the control group (*n* = 72). Analyses were adjusted for baseline height, body weight, baseline VO_2_max, and the corresponding baseline cognitive outcome. Panels show associations between VO_2_max improvement and changes in panel (a) 1-back accuracy, (b) 2-back accuracy, (c) 1-back reaction time, and (d) 2-back reaction time. Reaction-time improvement was calculated as pre-test minus post-test reaction time; thus, positive values indicate greater reductions in reaction time. None of the four partial correlations was statistically significant: 1-back accuracy, *r* = −0.071, *p* = 0.567; 2-back accuracy, *r* = 0.028, *p* = 0.822; 1-back reaction time, *r* = 0.074, *p* = 0.548; and 2-back reaction time, *r* = −0.101, *p* = 0.411. The Bonferroni-adjusted significance threshold was *p* < 0.0125.

Supplementary Table 1 Standardized 12-week HIIT schedule and three-stage progression.
