# Supplementary material for: Impact of a 12-week high-intensity interval training program on working memory in young adults with overweight or obesity
Source: Front Nutr. 2026 Sep 30;13:1869229. doi: 10.3389/fnut.2026.1869229 (PMC13661350; doi:10.3389/fnut.2026.1869229)
Supplement: Supplementary file 1 [file Table_1.docx]

| **Stage** | **Weeks** | **Frequency** | **Warm-up (10 min)** | **Calisthenic movement sequences (15 min)** | **Varied endurance running (15 min)** | **Fitness-based games (10 min)** | **Progression principle** |
| --- | --- | --- | --- | --- | --- | --- | --- |
| Stage 1: Familiarisation | 1–4 | Three non-consecutive sessions/week | Three minutes of jogging at 6–7 km/h (50–60% HRmax), followed by 4 min of joint mobilization involving the neck, shoulders, elbows, wrists, hips, knees, and ankles (15 repetitions each), and 3 min of dynamic stretching (2 sets of 10 repetitions). | Three 4-min sets of 30-s work/10-s rest, separated by 1-min rest. Approximately 60% of content involved low-impact modified movements, including step-back burpees without a jump, controlled bodyweight squats, standing torso rotations with knee-to-elbow contact, marching with arm movements, and modified high-knee movements. Approximately 40% involved carefully supervised lower-volume jump-based actions, such as low-amplitude tuck-jump variations or standard burpee variations where tolerated. | Five 40-s work/20-s slow-walking intervals, followed by 1-min slow-walking recovery. Approximately 60% of content involved brisk walking, fast marching, side stepping, controlled directional walking, and circular walking routes. Approximately 40% involved short-distance, individually tolerable S-cone slalom running, shuttle running, circular running, or group-based chase running. | Three 20-s work/10-s rest high-intensity segments. Approximately 60% of activities involved low-impact target-reaching, ball passing while marching, cone-reaching, plank exercises, and walking-based relay tasks. Approximately 40% involved short-distance shuttle-running or obstacle-course tasks. | Familiarisation with interval structure, correct movement technique, controlled landing, and individual tolerance. Running and jumping volume was deliberately limited. |
| Stage 2: Progressive overload | 5–8 | Three non-consecutive sessions/week | Same warm-up structure as Stage 1. | Three 4-min sets of 30-s work/10-s rest, separated by 1-min rest. Approximately 60% of content remained low-impact, including step-back burpees, controlled squat-based movements, standing core movements, and high-knee marching. The remaining 40% included an increased volume of standard burpees, tuck jumps, or high-knee movements with under-leg claps where appropriate. | Six to eight 40-s work/20-s slow-walking intervals, with 1-min slow-walking recovery after every five intervals. Approximately 60% of content remained low-impact locomotor activity. The remaining 40% involved increased short-distance running pace, slalom-running complexity, shuttle-running distance, and circular-running continuity. | Three 20-s work/10-s rest high-intensity segments. Low-impact game content remained approximately 60%, while the remaining 40% involved progressively longer shuttle-running, obstacle-course, and team chase tasks. | Workload was increased through greater repetition volume, movement continuity, running distance, stepping speed, route complexity, and task demands while retaining approximately 60% low-impact content. |
| Stage 3: Consolidation | 9–12 | Three non-consecutive sessions/week | Same warm-up structure as Stage 1. | Three 4-min sets of 30-s work/10-s rest, separated by 1-min rest. Standard burpees, tuck jumps, torso rotations with knee-to-elbow contact, and high knees with under-leg claps were performed with greater continuity and repetition volume. Low-impact modifications remained available when participants could not maintain safe technique. | Eight to ten 40-s work/20-s slow-walking intervals, with 1-min slow-walking recovery after every five intervals. S-cone slalom running, shuttle running, circular interval running, and group-based chase running were performed with greater distance, pace, and route complexity. Walking-based or stepping modifications remained available if required. | Three 20-s work/10-s rest high-intensity segments. Obstacle-course running, plank exercises, shuttle running, target tasks, and cooperative group activities were performed with increased movement continuity, task complexity, and participation intensity. | The highest planned workload was achieved through increased continuity, repetition volume, pace, running distance, directional-change demands, and game complexity. Movements were modified if participants experienced joint discomfort, dizziness, unusual shortness of breath, or inability to maintain correct technique. |
| All stages | 1–12 | Three non-consecutive sessions/week | 10 min | 15 min | 15 min | 10 min | Each session lasted 60 min, comprising a 10-min warm-up, 40-min core HIIT training, and 10-min cool-down. The cool-down included 2 min of slow walking to reduce heart rate to <50% HRmax, 6 min of static stretching with controlled abdominal breathing, and 2 min of box breathing. Six randomly selected participants wore heart-rate monitors during each session. Mean heart rate during high-intensity intervals was targeted at ≥85% HRmax, with RPE used to support intensity adjustment. |
